# A Nutritional and Anthropometric Analysis of the Double Burden of Malnutrition in Children Under Two in Madagascar

**DOI:** 10.3390/children12050640

**Published:** 2025-05-15

**Authors:** Rosita Rotella, María Morales-Suarez-Varela, Agustín Llopis-Gonzalez, Jose M. Soriano

**Affiliations:** 1 Research Group in Social and Nutritional Epidemiology, Pharmacoepidemiology and Public Health, Department of Preventive Medicine and Public Health, Food Sciences, Toxicology and Forensic Medicine, Faculty of Pharmacy and Food Sciences, Universitat de València, Av. Vicent Andrés Estelles s/n, 46100 Burjassot, Valencia, Spain; rotella@alumni.uv.es (R.R.); agustin.llopis@uv.es (A.L.-G.); 2 Centro Medico-Chirurgico Saint Paul (Change ONG), Andasibe-Ampefy 118, District de Soavinandriana, Itasy, Madagascar; 3 Biomedical Research Center in Epidemiology and Public Health Network (CIBERESP), Carlos III Health Institute, Av. Monforte de Lemos 3-5 Pabellón 11 Planta 0, 28029 Madrid, Spain; 4 Observatory of Nutrition and Food Safety for Developing Countries, Food & Health Lab, Institute of Materials Science, Universitat de València, Carrer Catedrático Agustín Escardino 9, 46980 Paterna, Valencia, Spain; jose.soriano@uv.es; 5 Joint Research Unit on Endocrinology, Nutrition and Clinical Dietetics, University of Valencia-Health Research Institute La Fe, Avda. Fernando Abril Martorell, 106, 46026 Valencia, Valencia, Spain

**Keywords:** double burden of malnutrition, Nutrimetry, nutritional status, Madagascar, child development

## Abstract

**Background**: Almost half of all deaths worldwide in children under five are related to malnutrition. Malnutrition encompasses a wide array of nutritional conditions and emerging evidence indicates a growing overlap of these different forms of malnutrition. Nutrimetry, which combines assessments of height-for-age (HAZ) with BMI-for-age (BMIZ) to offer a more integrated assessment of nutritional status, can be particularly useful in low-resource settings to correctly reflect the complex interplay of stunting and overweight. **Objective**: The objective of this study is to explore the impact of malnutrition on children in Madagascar and demonstrate how integrating HAZ assessments with BMIZ can reveal the double burden of malnutrition—encompassing both undernutrition and overnutrition—within the same population. **Methods**: This cross-sectional observational study employing Nutrimetry was carried out in rural communities in the Itasy region of Madagascar. A systematic random sampling method was used to choose the 500 women to invite to participate from the approximately 5000 who formed the pool of potential participants. A total of 437 were able to be invited and all invited women agreed to participate, resulting in 437 mother–child (0–24 months) pairs being included in the study. **Results**: Chronic undernutrition or thinness (31.6%), overweight and obesity (21.3%), and stunting (57.6%) were prevalent among the children included in the study. Among children with chronic undernutrition, 55.06% were identified as stunted. Among children with overweight or obese, 61.03% were identified as stunted. This highlights a significant overlap between inadequate weight and stunting. A socioeconomic analysis revealed significant barriers, including limited financial resources and poor dietary diversity, exacerbating malnutrition. Maternal nutritional status and breastfeeding practices also emerged as critical determinants of child nutritional outcomes. **Conclusions**: The study underscores the importance of prioritizing height assessments as a preliminary step in nutritional evaluations to prevent undetected acute malnutrition.

## 1. Introduction

Malnutrition, defined as an imbalance in the intake of energy or nutrients, encompasses undernutrition, overnutrition, and micronutrient deficiencies. Globally, approximately 144 million (21%) children under five suffer from stunting, a key indicator of chronic undernutrition [1]. Additionally, 45% of deaths among children under five are linked to undernutrition, particularly in low- and middle-income countries (LMICs), where childhood obesity rates are concurrently rising. Optimizing nutrition during the critical window of the first 1000 days of a child’s life—from conception to a child’s second birthday—is vital. Appropriate breastfeeding practices during this period significantly enhance the likelihood of optimal child development [2].

Traditionally, malnutrition has been studied in isolated dimensions, focusing separately on undernutrition, food insecurity, micronutrient deficiencies, or overweight and obesity [3]. However, emerging evidence indicates a growing overlap of different forms of malnutrition within individuals, families, and populations, termed the double burden of malnutrition (DBM) [4]. DBM describes the coexistence of undernutrition (including stunting, wasting, and micronutrient deficiencies) with overweight, obesity, and non-communicable diseases linked to diet [5].

Anthropometry, the measurement of human body dimensions, has been a cornerstone in assessing nutritional status, particularly in detecting malnutrition. However, despite its widespread use, the limitations of current anthropometric methods hinder their effectiveness in identifying chronic malnutrition, with important implications for public health. These limitations include inaccuracies in data quality [6], overreliance on proxy measures [7,8], missed cases of malnutrition [9,10,11,12], and challenges in special populations [7,8,13,14,15,16].

The limitations of current anthropometry measurements have significant implications for public health. The underestimation of malnutrition prevalence due to the limitations of anthropometric measures can lead to inadequate resource allocation and ineffective intervention programs [7]. The failure to detect malnutrition in its early stages can result in long-term consequences for child development, including stunted growth, cognitive impairments, and increased susceptibility to infections [12,15]. The underestimation of malnutrition in certain populations can lead to inequitable distribution of resources [12]. The limitations of anthropometric measures make it difficult to accurately monitor the impact of nutrition interventions. This can lead to incorrect conclusions about the effectiveness of programs and hinder efforts to improve nutrition outcomes [17]. The failure to detect malnutrition early can result in more severe health complications, leading to increased healthcare costs [13]. The limitations of anthropometric measures can lead to poorly informed policy decisions [9,10,11,12].

In 2023, an authoritative report highlighted Madagascar as one of the countries that is most severely affected by malnutrition, with rates worsening significantly between 2020 and 2022 [18]. Nationally, 64.9% of households experienced food insecurity during this period, with the Itasy region reporting a stunting prevalence of 62.3%, peaking at 18–23 months of age [18].

Malnutrition during critical developmental periods—such as prenatal life, infancy, and early childhood—has profound and lasting impacts, increasing the risk of chronic diseases and reducing overall health and socioeconomic potential in adulthood. Both prenatal and early childhood malnutrition are strongly linked to increased risks of cardiometabolic diseases [19,20,21,22,23], impaired organ function [19,24,25], and immune system impairment [26]. The timing of malnutrition is crucial. Early gestation and the first three years of life are particularly vulnerable periods, with long-lasting effects on organ structure, metabolism, and disease risk [19,27]. Malnutrition can cause epigenetic changes and alter developmental pathways, affecting organ growth, immune function, and metabolic regulation without necessarily changing birth size [19,21,24]. Deficits in high-quality protein, zinc, and other micronutrients during recovery from malnutrition can limit lean tissue growth and further increase chronic disease risk [21,26]. Survivors of early malnutrition often experience diminished intellectual performance, lower work capacity, and reduced socioeconomic status in adulthood [23,27]. Malnutrition is linked to delayed cognitive and behavioural development, which can have lifelong consequences [27]. Early prevention and adequate nutrition during these windows are essential for lifelong health.

Stunting, defined as a height-for-age less of than two standard deviations below the WHO child growth standard median, has long-term consequences for physical and cognitive development, contributing to behavioural disorders in adulthood and adversely affecting national economic growth [28,29]. Furthermore, stunted individuals exhibit a heightened risk of obesity later in life due to altered metabolic profiles, including lower resting energy expenditure and reduced fat oxidation, which predispose them to weight gain [30,31].

The DBM is a growing public health issue in low- and middle-income countries (LMICs) [32,33]. Prevalence varies by age group, country, and socioeconomic status, but evidence shows that the double burden is common and increasing, especially among children and adolescents [32,33]. Among children under 5 years in LMICs, the prevalence of concurrent stunting and overweight at the individual level ranges from about 0.7% to 10.9%, with 0.6% to 37.8% of stunted children also being overweight in some countries [34,35]. Nationally, only a small number of countries (5 out of 93) meet the World Health Organization’s threshold for double burden at the national level, but about one-third of countries show higher-than-expected individual-level prevalence [35].

Over the past two decades, stunting and wasting have generally decreased, while overweight and obesity have increased in many LMICs [5,34,36]. The problem is shifting from primarily affecting poor households to also impacting wealthier households in poorer countries and poorer households in wealthier LMICs [5,37]. Economic and social globalisation, urbanisation, and changes in food systems (e.g., increased availability of ultra-processed foods) are linked to higher double burden prevalence, particularly among the poorest populations [5,37]. Risk factors include food insecurity, diet behaviour, breastfeeding practices, illness, metabolic programming, household wealth, women’s education, and urbanisation [38].

The context of maternal and child nutrition during the first 1000 days in Madagascar is critical, as this period significantly influences health outcomes for both mothers and children. This timeframe, encompassing pregnancy through the first two years of a child’s life, is essential for growth and development. In Madagascar, maternal nutritional status is often compromised, leading to adverse effects on foetal growth and infant health. A high prevalence of maternal overweight and obesity, alongside undernutrition, is observed in Madagascar [39]. Also, maternal anaemia is common, negatively impacting birth weight and increasing infant mortality risk [39]. When it comes to the nutrition challenges in children, the reliance on low-nutrient cereal-based diets contributes to inadequate dietary diversity, exacerbating malnutrition [40]. Stunting, a significant concern, is linked to chronic malnutrition during the first 1000 days [41]. Despite these challenges, there is a growing recognition of the importance of addressing maternal and child nutrition in Madagascar. However, the effectiveness of interventions may vary based on local contexts and resources, necessitating tailored approaches to improve outcomes.

The area of study, a rural municipality of the Itasy region of Madagascar, is characterised by a subtropical climate influenced by monsoon patterns. During the rainy season (November–March), destructive floods disrupt agricultural production and exacerbate food insecurity, while the dry season (May–September) poses challenges due to prolonged periods without rainfall. Madagascar ranks among the nation’s most vulnerable to the impacts of the global climate crisis [42,43].

This study aims to evaluate the DBM at the individual level in the Itasy region of Madagascar using the innovative Nutrimetry methodology [44]. This approach combines two anthropometric measures—BMI-for-age (BMIZ) and height-for-age (HAZ)—to facilitate an integrated interpretation of nutritional status. By addressing the limitations of conventional BMI assessments, Nutrimetry provides a comprehensive view of how stunting and obesity coexist and interact.

## 2. Materials and Methods

### 2.1. Study Design and Population

The exploratory, observational, and analytical study conducted between 1 November 2022 and 31 March 2023 targeted women residing in Ampefy who had children aged 0–24 months. Ampefy has a population of 25,078, divided into 13 administrative units known as fokontany, the smallest administrative divisions in Madagascar. The population is ethnically diverse, with the Merina ethnic group being predominant.

### 2.2. Study Population and Data Collection

The study population was identified through the patient registry of St. Paul’s Medical and Surgical Centre, which collaborates with the NGO Change Onlus to support maternal and child health initiatives in the Itasy region. Women meeting the following inclusion criteria were considered eligible: (i) aged 15–49 years (reproductive age), (ii) having at least one living child aged 0–24 months, and (iii) currently breastfeeding at least one child in this age range. Exclusion criteria included the following: (i) refusal to provide informed consent, (ii) unreliable responses during data collection, and (iii) incomplete responses to the questionnaire.

Eligible participants were selected using a systematic random sampling method from the health centre registers. Women attending the health centre with their children, or those accessing services during the days when project personnel were present in the fokontany, were invited to participate. The demographic data of Ampefy municipality indicate that approximately 5000 women of reproductive age (12–49 years) reside in the region. Among these, 437 women were systematically invited to participate, all of whom provided informed consent, resulting in a 100% response rate and the inclusion of 437 mother–child pairs.

Recruitment and data collection spanned from November 2022 to March 2023. The research team underwent a comprehensive seven-day training program to standardize data collection techniques and ensure quality control. The training included the use of the questionnaire and anthropometric measurement tools, as well as piloting study procedures with a subset of participants who were not included in the final study.

All participants were informed of the study’s objectives and assured of the confidentiality and anonymity of their data. Consent forms were translated orally by health centre staff to ensure that the participants understood the study’s purpose and their rights. To maintain anonymity, each participant was assigned a unique identification number. Data collection was supervised by the project coordinators to ensure adherence to protocols and to address any issues that arose during fieldwork.

Data collection took place in the 13 fokontany of Ampefy and at the NGO’s headquarters, the health centre. A structured questionnaire was administered through face-to-face interviews conducted in the local language by trained staff, with each interview lasting approximately 20 min to collect a range of information, including maternal medical history, sociodemographic characteristics, dietary practices, access to healthcare services, and breastfeeding behaviours. Maternal medical histories were reviewed when available in the health centre records to cross-validate self-reported data. Tablets were used to record responses directly, minimizing transcription errors.

Anthropometric measurements for both mothers and children were taken at the same time as the interview was conducted and recorded using standard techniques. Child length (for children under 12 months) or height (for children 12 months and older) were measured with a precision stadiometer, while maternal height was measured with the same equipment. Child weight was recorded using a calibrated electronic baby scale, and maternal weight was assessed with an adult electronic scale. Mid-upper arm circumference (MUAC) was measured for mothers and children using non-stretchable measuring tapes. The anthropometric data were used to calculate BMIZ and HAZ based on WHO growth standards.

### 2.3. Ethical Considerations

The study adhered to the World Medical Association’s Declaration of Helsinki (2000) on Ethical Principles for Medical Research, as clarified in 2002 and 2004 [45]. It also followed the Guidelines on the Practice of Ethics Committees Involved in Medical Research and the Ethical Conduct of Medical Research Involving Children [46]. Ethical approval was obtained from The Ethics Committee of the University of Valencia, Spain (protocol code: 2089516, approval date: 7 July 2022) and The Ethics Committee of St. Paul Medical-Surgical Centre and the Institut d’Enseignement Supérieur de Soavinandriana, Université d’Antananarivo, Madagascar (protocol code: 20220197, approval date: 27 October 2022). Informed consent, written or verbal, was obtained from all participants. Verbal consent was witnessed and formally documented.

### 2.4. Measurement Tools

The semi-structured, non-pretested questionnaire was specifically designed to collect data on maternal education, attitudes toward antenatal care, dietary habits during pregnancy and lactation, and the relationship between micronutrient intake, dietary diversity, and sociodemographic factors. Initially drafted in English, the questionnaire was translated into Malagasy by a local translator fluent in English. A second translation ensured accuracy and preserved the content’s integrity. To verify its usability, ten mothers from the health centre reviewed the questionnaire and provided feedback.

After administering the pilot questionnaire to the 10 mothers, attention span limitations were observed after 25 min. Consequently, the questionnaire was revised, removing questions about personal hygiene and paternal influence on feeding decisions. The number of items in the questionnaire was reduced from 60 to 43. Mothers in the pilot study were excluded from the final sample to avoid bias.

The final questionnaire consisted of four sections: (i) maternal characteristics (age, education, occupation, and pregnancies); (ii) practices during pregnancy and breastfeeding (antenatal care, place of birth, breastfeeding); (iii) living conditions (health status, water access, proximity to health centres, and transport availability); and (iv) an assessment of Minimum Dietary Diversity for Women of Reproductive Age (MDD-W) [47].

Dietary intake during the previous 24 h was assessed using a list-based technique, where interviewers presented a culturally adapted food list reflecting the ten food groups defined by FAO and FHI 360’s MDD-W [48]. Mothers provided detailed descriptions of foods and ingredients consumed within the household, which were categorised based on their composition. Data collectors were trained to classify meals containing mixed food groups, recording only those where consumption exceeded 15 g per group.

Maternal measurements were obtained using a SECA stadiometer (Hamburg, Germany; precision: 0.1 cm) and a SECA Clara 803 electronic scale (Hamburg, Germany; precision: 0.1 kg). Two height measurements were taken, and the average was used to minimise errors. BMI was calculated by dividing weight by height squared (kg/m^2^), with nutritional status classified as underweight, normal weight, overweight, or obese according to WHO cut-off values [28].

Anthropometric assessments were performed by experienced interviewers, each with over seven years of involvement in the Child Malnutrition Prevention and Treatment Project by Change Onlus. For children under 12 months, length was measured in the prone position using UNICEF length charts (precision: 0.1 cm). For children older than 12 months, height was measured standing with equivalent precision. A trained nutritionist assisted during measurements to ensure reliability. Nutritional status was determined using WHO cut-off points for weight-for-height (WFH), HFA, weight-for-age (WFA), and BMIZ [28]. All raw data were entered into WHO AnthroPlus 1.0.4 software for calculation [49], except WFH, which was determined using an online tool.

Additionally, Nutrimetry [44] was employed to assess nutritional status by combining BMIZ and HAZ into a single framework. This method uses Nutricodes, a system of nine diagnostic codes (1, 3, 4, 5, 6, 7, 8, 9, 11) displayed in a 3 × 3 matrix for conceptual interpretation. The Nutricodes are defined as follows: 1 (Low HAZ + Low BMIZ), 3 (Normal HAZ + Low BMIZ), 4 (Low HAZ + Normal BMIZ), 5 (High HAZ + Low BMIZ), 6 (Normal HAZ + Normal BMIZ), 7 (High HAZ + Low BMIZ), 8 (High HAZ + Normal BMIZ), 9 (Low HAZ + High BMIZ), and 11 (High HAZ + High BMIZ). Nutricodes differentiate nutritional statuses: even numbers (4, 6, 8) represent healthy weight, smaller odd numbers (1, 3, 5) indicate thinness, and larger odd numbers (7, 9, 11) denote overweight or obesity. Numbers 3, 6, and 9 reflect normal height, while 1, 4, and 7 indicate short stature, and 5, 8, and 11 signify tall stature. This approach effectively captures the dual burden of malnutrition and highlights the long-term risks of stunting related to overweight and obesity.

### 2.5. Data Analysis

Continuous variables were presented as means with standard deviations (SDs) or medians with interquartile ranges (IQRs), depending on the distribution of the data, while categorical variables were expressed as frequencies and percentages. The Kolmogorov–Smirnov test was applied to evaluate the normality of data distribution.

Comparisons between stunted and non-stunted children were performed using appropriate statistical tests: the Chi-square test or McNemar test for categorical variables and analysis of variance (ANOVA) for continuous variables. Statistical significance was determined at a threshold of α < 0.05.

All statistical analyses were conducted using IBM SPSS Statistics version 26.0 (IBM Corp., Armonk, NY, USA). The software facilitated comprehensive data evaluation, ensuring accurate interpretation of results and adherence to rigorous statistical standards.

## 3. Results

### 3.1. Baseline Characteristics of the Infants

The study included 437 mother–child pairs. Anthropometric measurements were used to calculate the HAZ and BMIZ for the children. These scores were subsequently integrated into nine categories, referred to as Nutricodes, to reflect distinct nutritional statuses (Table 1).

For thinness (BMIZ ≤ −1), groups 1, 3, and 5 represent children classified as experiencing ‘Thinness’ according to Nutrimetry. These groups collectively account for 31.6% of the sample (Table 1). Among these children, 55.06% were identified as stunted (HAZ ≤ −2), highlighting a significant overlap between thinness and stunting. For healthy weight (BMIZ between −1 and +1), groups 4, 6, and 8 correspond to children categorised as having a ‘Healthy Weight’ by Nutrimetry. This group represents 47.1% of the total sample. Within this group, 57.37% of the children were stunted, underscoring the prevalence of linear growth retardation even among those classified as having normal weight. For overweight or obese (BMIZ ≥ +1), groups 7, 9, and 11 include children classified as ‘Overweight or Obese’ by Nutrimetry. These groups constitute 21.3% of the sample. Notably, 61.03% of these children were stunted, indicating that excess weight often coexists with chronic undernutrition in this population.

These findings demonstrate a high prevalence of stunting across all Nutrimetry categories, emphasizing the critical need for height assessments alongside weight-based evaluations to accurately capture the dual burden of malnutrition in children.

### 3.2. Characteristics of the Children

Table 2 summarizes the anthropometric and nutritional characteristics of the infants in the study. The mean age of the children was 10.53 ± 6.48 months, with significant differences observed across Nutricode categories. The Healthy Weight category was most prevalent among children aged 14–24 months, whereas the Thinness category was also common in this age group. Conversely, the Overweight/Obesity category was more frequent among infants aged 0–6 months. No significant differences in sex distribution were identified across groups. Low birth weight (LBW, defined as <2.5 kg) was observed in 20.6% of the sample, with the highest prevalence in the Thinness category: 35.5% in Group 1, 18.5% in Group 3, and 25.0% in Group 5. LBW was less common in the Healthy Weight and Overweight/Obesity categories, with significant differences between these classifications. The mean weight was 7.31 ± 1.74 kg, and the mean height was 66.20 ± 8.52 cm, both showing significant variations across Nutricode groups. 

Severe acute malnutrition (MUAC < 114 mm) was observed in 2.6% of the sample, predominantly in Group 1 (11.6%), and was absent in other categories. Moderate acute malnutrition (MUAC 115–124 mm) affected 19.4% of the sample, primarily in the Thinness category (56.5% in Group 1, 22.2% in Group 3, and 14.3% in Group 5). Minimal cases were noted in the Healthy Weight group, with no instances in the Overweight/Obesity category. The mean weight-for-length (WFL) Z-score was −0.01 ± 1.85, with significant intergroup differences. Moderate and severe malnutrition (WFL Z-score below −2) occurred mainly in Groups 1, 3, and 5, whereas elevated WFL values (above +2 Z-score) were most frequent in Groups 7 and 9. The mean WFA Z-score was −1.47 ± 1.42, varying significantly across groups. Higher WFA Z-scores (above +1 SD) were more common in Groups 7, 9, and 11, suggesting weights exceeding international norms for age. The HFA analysis revealed severe stunting prevalence in Groups 1, 4, and 7, with rates of 46.1%, 45.4%, and 70.2%, respectively. Moderate stunting affected 27.9% of the total sample, reflecting a substantial burden of chronic undernutrition in the study population.

### 3.3. Characteristics of the Mothers

The maternal profiles summarized in Table 3 showed almost no significant differences across groups, reflecting the homogeneity of the rural study population. Mothers of children in the Thinness category had the lowest mean weight (45.60 ± 7.10 kg) and were shorter (151.14 ± 5.20 cm), whereas mothers of children in the Overweight/Obesity category were the heaviest (51.87 ± 8.60 kg). Maternal BMI differed significantly among groups. Underweight mothers (BMI < 18.5) represented 24.7% of the sample, most commonly in the Thinness group (36.2%). Normal BMI (18.5–24.9) was observed in 66.1% of mothers, predominantly in the Healthy Weight group. Overweight (BMI 25.0–29.9) was recorded in 7.8% of mothers, mainly in the Overweight/Obesity group (11.6%). Obesity (BMI ≥ 30) was rare, affecting only 1.4% of mothers, with a slight predominance among mothers of thin children.

### 3.4. Maternal Care and Feeding Practices

Table 4 summarises maternal care and feeding practices according to the Nutricode classifications of their children. Delivery methods showed significant variation among groups. Vaginal delivery was the most common mode of delivery (92.0%), particularly in the Thinness group (97.4%). Operative vaginal deliveries accounted for 5.3%, with the highest prevalence in the Overweight/Obesity group (8.6%). Caesarean sections were infrequent (2.7%) but more common in the Thinness group (5.1%), where seven out of the 12 caesarean deliveries occurred. Most deliveries (64.1%) took place in health centres, while 35.9% occurred at home, with the Thinness group having the highest proportion of home births (38.2%).

Maternal dietary practices during pregnancy and breastfeeding exhibited notable differences. While 53.8% of mothers reported improving their diet during pregnancy, this was less frequent in the Thinness group (39.5%) compared to the Healthy Weight (55.3%) and Overweight/Obesity (61.3%) groups. During breastfeeding, 41.6% of mothers reported dietary improvements, with the highest proportion in the Overweight/Obesity group (44.1%). 

### 3.5. Socioeconomic Characteristics of Households

Table 5 summarises the socioeconomic characteristics of the households in the study. There are no observed differences between the groups, but the data reflect the economic and infrastructural challenges faced by households with a consistently high dependency ratio, limited living space and overcrowding. Low–middle income, defined as a monthly income below MGA Ar 200,000, was reported by 75.1% of households. Food insecurity was a significant concern, as 32.0% of households lacked sufficient rice for at least six months of the year. Land ownership, crucial for agricultural and financial stability, was reported by 51.7% of households, with comparable levels across groups. Protected wells were the primary water source for 65.2% of households and access to latrines was widespread, with 91.1% of households reporting availability. Transportation challenges were reported by 25.9% of households, and the average walking time to health centres was 47.67 ± 33.04 min.

### 3.6. Dietary Diversity of the Mothers

Table 6 summarises the dietary diversity of mothers, evaluated using the Minimum Dietary Diversity for Women (MDD-W) questionnaire, in relation to their child’s Nutricode classification. The findings reveal that 33.0% of mothers consumed fewer than five food groups, reflecting limited dietary diversity. Despite some variation in individual food group consumption, no statistically significant differences were observed in overall dietary diversity across Nutricode groups. Reliance on staple foods such as grains and tubers was uniform, highlighting their central role in the diet. However, the limited intake of animal proteins (e.g., eggs and dairy) suggests potential economic constraints or cultural dietary preferences. The overall dietary diversity remains insufficient for many mothers, underscoring the need for improved dietary interventions to enhance nutritional adequacy in this population.

## 4. Discussion

This study offers valuable insights into the anthropometric measurements, socioeconomic characteristics, maternal care practices, and dietary diversity of mothers in relation to their child’s Nutricode classification. Groups 1 (Low HAZ + Low BMIZ), 3 (Normal HAZ + Low BMIZ), and 5 (High HAZ + Low BMIZ) contained a significant number of children, while the highest prevalence was found in groups 4 (Low HAZ + Normal BMIZ), 5 (High HAZ + Low BMIZ), 1 (Low HAZ + Low BMIZ), and 3 (Normal HAZ + Low BMIZ). The results underscore the complex interplay between maternal and child nutrition, socioeconomic factors, and health practices.

### 4.1. Anthropometry and Nutritional Status of Children

The data revealed substantial variability in BMIZ and HAZ among children. Of the participants, 31.6% were classified as thin, 21.3% as overweight or obese, and 47.1% had a healthy weight. These findings underscore the dual burden of malnutrition in LMICs, where undernutrition and overnutrition coexist. Similar studies in Colombia and Malaysia also highlight the importance of addressing this dual burden through comprehensive nutritional assessments and early interventions [50,51].

The use of nutrimetry, which combines BMIZ and HAZ to offer a more integrated assessment of nutritional status, proved valuable in identifying at-risk children. This method is particularly relevant in low-resource settings, where traditional assessments may fail to capture the complex interplay of stunting and overweight. Stunting’s long-term effects, including an increased risk of obesity and associated complications such as cardiovascular disease and hypertension, further emphasize the need for early detection and prevention strategies.

As a methodology for nutritional assessment, nutrimetry is still in its infancy and has only been applied in limited situations previously [44,52,53,54,55,56,57]. It has been used in paediatric populations in Mexico (0–19 and 5–11 year olds) [44,55], Spain (3–11, 6–9 year olds, and 0–30 month olds) [52,54,57], Ecuador (3–11 year olds) [53], and Madagascar [56]. The study conducted in Madagascar was on children aged 6 to 17 years old and therefore is not comparable to the population of this study [56]; the only study conducted on a population of similar age (under two years old) was conducted in Spain [57], which is a developed country and is therefore also not comparable.

### 4.2. Maternal Profile and Its Influence on Child Nutrition

The average maternal age was 25.75 years, with 66.1% of mothers within the normal BMI range. However, 24.7% were underweight, with the highest prevalence in the Thinness group (36.2%), highlighting the link between maternal and child nutritional status. These findings support evidence on the intergenerational transmission of malnutrition, where undernourished mothers are more likely to give birth to underweight infants, predisposing them to stunting and other developmental challenges [58,59].

Adequate maternal nutrition, particularly during pregnancy, is critical for preventing low birth weight and ensuring optimal foetal growth. Deficiencies in iron and folate during pregnancy can lead to adverse outcomes such as preterm birth and impaired foetal development [60,61]. Additionally, maternal malnutrition exacerbates the risk of long-term health issues in children, including cognitive impairments and chronic diseases [62]. Addressing socioeconomic factors such as poverty and healthcare access is imperative to breaking this cycle of intergenerational malnutrition [63].

### 4.3. Maternal Care Practices

Antenatal care (ANC) coverage was high, with over 90% of mothers attending at least four visits and 83.8% receiving iron and folic acid supplements. These practices are essential for improving maternal and foetal health outcomes [64]. However, caesarean sections were more common in the Thinness group, consistent with evidence linking maternal nutritional deficiencies to pregnancy complications requiring surgical intervention [65,66].

While breastfeeding rates were high, with 96.1% of mothers breastfeeding during the first year, early weaning was prevalent, reported by 60.9% of mothers. Early weaning may compromise infant nutrition and increase susceptibility to infections and malnutrition [67], underlining the need for targeted education and support for optimal breastfeeding practices.

### 4.4. Socioeconomic Factors

The average household size was 4.59 persons, with 48.3% of households living in overcrowded conditions. Low household income, reported by 75.1% of families, limits access to adequate nutrition and healthcare, contributing to food insecurity. Approximately 32.0% of households reported insufficient rice for at least six months of the year, a situation exacerbated in the Thinness (35.5%) and Overweight/Obesity (40.9%) groups.

Sociodemographic and cultural aspects in Madagascar significantly influence the development of DBM and contribute to varying nutritional and health outcomes. Additionally, the country’s diverse ecological contexts and socioeconomic disparities further complicate the nutritional landscape, leading to a complex interplay of factors that drive the DBM in Madagascar. Environmental changes, including deforestation and climate shifts, affect food production and availability, leading to nutritional deficiencies and increased disease burden [68]. Socioeconomic disparities, such as income inequality and access to resources, contribute to malnutrition, with some non-poor households experiencing stunting and some poor households having non-stunted children [69]. Cultural practices and dietary habits influence nutritional outcomes, with traditional diets sometimes lacking in essential nutrients, contributing to undernutrition [68]. Demographic transitions, including urbanisation and changes in lifestyle, contribute to the nutrition transition, where populations experience shifts from traditional diets to more processed foods, increasing the risk of overnutrition [70]. While Madagascar faces significant challenges in addressing the DBM, it is crucial to consider the broader implications of these issues. The DBM is not only a health concern but also an economic and social one, affecting future generations and the nation’s development. Addressing these challenges requires a holistic approach that integrates environmental, cultural, and socioeconomic factors into comprehensive nutrition policies and programs.

Economic constraints often lead to reliance on seasonal agriculture and cheaper, energy-dense foods, increasing the risks of both undernutrition and obesity [71,72,73]. These findings align with research linking low socioeconomic status to poor dietary diversity and adverse nutritional outcomes [18]. Limited access to healthcare further compounds the effects of malnutrition, as financial barriers prevent many households from seeking timely medical care [74]. Addressing these socioeconomic challenges is vital for improving nutritional outcomes and reducing the dual burden of malnutrition.

### 4.5. Dietary Diversity Among Mothers

Approximately 33% of mothers consumed fewer than five food groups, reflecting low dietary diversity, particularly in the Thinness group (39.5%). While grains and tubers were universally consumed, the intake of eggs (10.1%) and dairy products (29.3%) was notably low. This limited diversity indicates potential deficiencies in key nutrients such as calcium, vitamin D, and protein, all of which are essential for maternal and child health [75,76]. Mothers in the Healthy Weight and Overweight/Obesity groups reported higher consumption of animal proteins, such as meat, poultry, and fish, suggesting better access to these nutrient-rich foods, which contribute to improved dietary quality. However, the overall dietary diversity was constrained, particularly among low-income households. This highlights the need for interventions that promote diverse diets to reduce malnutrition risks and associated health complications [77]. Strategies could include nutritional education, food fortification, and policies that encourage the production, availability, and affordability of a broader range of food groups.

### 4.6. Applicability to Practice and Research

The significant prevalence of the double burden of malnutrition in children under two years old in Madagascar highlights critical areas for future practice and research. Addressing this issue requires a multifaceted approach that combines targeted interventions, improved food security, and tailored nutritional policies. These findings can inform future practices by emphasizing the importance of localized and context-specific strategies that consider the unique socioeconomic and geographical factors influencing malnutrition. The integration of these insights into policy and practice can lead to more effective resource allocation and intervention strategies. While these findings emphasize the need for targeted and context-specific interventions, it is also crucial to consider the broader socioeconomic and environmental challenges that may impact the implementation of these strategies. Factors such as political stability, infrastructure development, and climate change can significantly influence the success of nutritional interventions in Madagascar and addressing these may yield more sustainable solutions in the long term.

Longitudinal studies could provide insights into the long-term effects of interventions and help refine strategies to combat malnutrition effectively. Utilizing small-area estimation techniques can provide precise commune-level data on malnutrition, allowing for targeted interventions rather than broad, ineffective policies [69].

Investigating the interplay between poverty and malnutrition can inform more effective resource allocation and intervention strategies [69]. The prevalence of malnutrition in non-poor households suggests that interventions should not solely focus on poverty alleviation but also address nutritional education and access to quality foods across all socioeconomic groups [69]. Programs should focus on educating parents about the importance of a diversified diet for children aged 6-23 months, emphasizing the need for micronutrient-rich foods [78].

Understanding seasonal variations in food security can guide the development of agricultural strategies that enhance food self-sufficiency during lean periods, thereby reducing malnutrition rates [79]. Future research should explore seasonal dietary patterns and their impact on child malnutrition, providing insights for timely interventions [79].

Long-term strategies should aim to enhance household food self-sufficiency, particularly during lean periods when food insecurity peaks [79]. Ensuring that vulnerable populations have access to affordable, fortified foods is essential for combating malnutrition [78].

### 4.7. Strengths and Limitations

This study has several strengths, including the innovative application of Nutrimetry, which integrates BMI-for-age and height-for-age measurements to provide a comprehensive assessment of nutritional status. The large sample size of 437 mother–child pairs enhances the generalizability of the findings to similar rural settings. Rigorous data collection processes, including the use of structured questionnaires and trained professionals, ensured the reliability and accuracy of the data. However, the study’s cross-sectional design limits its ability to establish causality between variables. Self-reported data on maternal diet and socioeconomic status may introduce recall or social desirability biases. Additionally, single-point measurements for certain indicators might not fully capture variability over time, potentially affecting the reliability of some outcomes. Moreover, the sample may over-represent families with better access to healthcare services, potentially skewing the findings and limiting generalizability to less engaged or more disadvantaged populations. Addressing these limitations in future research, such as incorporating longitudinal designs or reaching underserved populations, could provide a more nuanced understanding of the dynamics between maternal and child nutrition.

## 5. Conclusions

This study highlights the DBM in Madagascar, where chronic malnutrition among children manifests as reduced height-for-age. This stunting complicates the accurate identification of malnourished children when using BMI as the primary anthropometric indicator, potentially leading to underdiagnosis and missed interventions. The findings also reveal an association between maternal characteristics and children’s nutritional status, emphasizing the intergenerational nature of malnutrition. The coexistence of undernutrition and overnutrition within the same population reflects the complex dynamics of malnutrition in low- and middle-income countries. Chronic undernutrition and stunting coexist with increasing risks of childhood obesity, illustrating the urgent need for integrated approaches to nutritional assessment and intervention. The study underscores the necessity of prioritising height assessments to determine whether children’s growth is appropriate for their age before conducting further anthropometric evaluations. This methodological adjustment can improve the identification of at-risk children and enable the implementation of targeted nutritional interventions, particularly during the critical first 1000 days of life. Addressing both undernutrition and childhood obesity within this window is essential for improving long-term health outcomes and breaking the cycle of intergenerational malnutrition.

## Figures and Tables

**Table 1:** Anthropometry of the infants by Nutricodes (n = 437).

	BMI/Age (BFA)	z ≤ −1 +0 Thinness n = 138 (31.6%)	−1< z < +1 +3 Healthy Weight n = 206 (47.1%)	z ≥ +1 +6 Overweight/Obesity n = 93 (21.3%)
Height/Age (HFA)				
**z ≥ +2** **+5** **High stature** n = 10 (2.2%)		8 (1.8%)	1 (0.2%)	1 (0.2%)
**−2 < z < +2** **+3** **Normal stature** n = 175 (40.2%)		54 (12.4%)	86 (19.7%)	35 (8.0%)
**z ≤ −2** **+1** **Low stature** n = 252 (57.6%)		76 (17.4%)	119 (27.2%)	57 (13.0%)

**Table 2:** Profile of the infants.

		NUTRICODE													
	Total N = 437	Thinness (n = 138, 31.6%)				Healthy Weight (n = 206; 47.1%)				Overweight/Obesity (n = 93; 21.3%)				*p*-Value ^1^	*p*-Value ^2^
	Frequency (%) Mean ± SD	1 (n = 76; 17.4%)	3 (n = 54; 12.4%)	5 (n = 8; 1.8%)	Total (n = 138, 31.6%)	4 (n = 119; 47.1%)	6 (n = 86; 19.7%)	8 (n = 1; 0.2%)	Total (n = 206; 47.1%)	7 (n = 57; 13.0%)	9 (n = 35; 8.1%)	11 (n = 1; 0.2%)	Total (n = 93; 21.3%)		
**Age**	10.53 ± 6.48	11.87 ± 5.39	9.66 ± 5.88	13.12 ± 7.22	11.08 ± 5.77	12.80 ± 5.85	9.26 ± 6.66	7.00	11.29 ± 6.42	8.78 ± 7.51	6.97 ± 6.07	3.00	8.04 ± 7.00	<0.001	<0.001
0–6 months	137 (31.4%)	13 (17.1%)	18 (33.3%)	1 (12.5%)	32 (23.2%)	20 (16.8%)	37 (43.0%)	0 (0%)	57 (27.7%)	28 (49.1%)	19 (54.3%)	1 (100%)	48 (51.6%)	<0.001	<0.001
7–13 months	148 (33.9%)	31 (40.8%)	23 (42.6%)	5 (62.5%)	59 (42.8%)	43 (36.1%)	24 (27.9%)	1 (100%)	68 (33.0%)	10 (17.5%)	11 (31.4%)	0 (0%)	21 (22.6%)		
14–24 months	152 (34.8%)	32 (42.1%)	13 (24.1%)	2 (25.0%)	47 (34.1%)	56 (47.1%)	25 (29.1%)	0 (0%)	81 (39.3%)	19 (33.3%)	5 (14.3%)	0 (0%)	24 (25.8%)		
Total n = 437	437 (100%)	76 (100%)	54 (100%)	8 (100%)	138 (100%)	119 (100%)	86 (100%)	1 (100%)	206 (100%)	57 (100%)	35 (100%)	1 (100%)	93 (100%)		
**Sex**														0.711	0.402
Female	217 (49.7%)	31 (40.8%)	27 (50.0%)	4 (50.0%)	62 (44.9%)	61 (51.3%)	44 (51.2%)	1 (100%)	106 (51.5%)	28 (49.1%)	20 (57.1%)	1 (100%)	49 (52.7%)		
Male	220 (50.3%)	45 (59.2%)	27 (50.0%)	4 (50.0%)	76 (55.1%)	58 (48.7%)	42 (48.8%)	0 (0%)	100 (48.5%)	29 (50.9%)	15 (42.9%)	0 (0%)	44 (47.3%)		
Total n = 437	437 (100%)	76 (100%)	54 (100%)	8 (100%)	138 (100%)	119 (100%)	86 (100%)	1 (100%)	206 (100%)	57 (100%)	35 (100%)	1 (100%)	93 (100%)		
**Low birth weight ^3^**	90 (20.6%)	27 (35.5%)	10 (18.5%)	2 (25.0%)	39 (28.3%)	26 (21.8%)	12 (14.0%)	0 (0%)	38 (18.4%)	13 (22.8%)	0 (0%)	0 (0%)	13 (14.0%)	0.004	0.036
**Weight (kg)**	7.31 ± 1.74	6.49 ± 1.37	6.94 ± 1.37	8.53 ± 1.58	6.78 ± 1.45	7.50 ± 1.31	7.65 ± 2.02	8.70	7.56 ± 1.64	7.83 ± 2.30	7.83 ± 2.30	7.31	7.51 ± 2.14	<0.001	<0.001
**Height (cm)**	66.20 ± 8.52	66.42 ± 6.60	69.06 ± 7.43	79.50 ± 6.05	68.21 ± 7.52	67.10 ± 6.83	67.44 ± 9.11	73.00	67.26 ± 7.84	59.88 ± 9.30	62.60 ± 9.14	57.00	60.87 ± 9.24	<0.001	<0.001
**MUAC** **(mm)**	135.91 ± 11.72	124.61 ± 9.15	133.47 ± 8.10	138.30 ± 12.08	128.69 ± 10.19	135.54 ± 9.33	142.27 ± 10.11	130.00	137.91 ± 10.11	142.50 ± 8.08	152.53 ± 9.79	-	146.24 ± 9.95	<0.001	0.001
MAS ≤114 mm	9 (2.6%)	8 (11.6%)	0 (0%)	0 (0%)	8 (6.6%)	1 (0.9%)	0 (0%)	0 (0%)	1 (0.6%)	0 (0%)	0 (0%)	-	0 (0%)	<0.001	<0.001
MAM ≥115 mm − ≤124 mm	67 (19.4%)	39 (56.5%)	10 (22.2%)	1 (14.3%)	50 (41.3%)	16 (14.4%)	1 (1.6%)	0 (0%)	17 (9.8%)	0 (0%)	0 (0%)	-	0 (0%)		
Normal ≥ 125 mm	270 (78.0%)	22 (31.9%)	35 (77.8%)	6 (85.7%)	63 (52.1%)	94 (84.7%)	61 (98.4%)	1 (100%)	156 (89.7%)	32 (100%)	19 (100%)	-	51 (100%)		
Total n = 346	346 (100%)	69 (90.8%)	45 (83.3%)	7 (87.5%)	121 (87.7%)	111 (93.3%)	62 (72.1%)	1 (100%)	174 (84.5%)	32 (56.1%)	19 (54.3%)	-	51 (54.8%)		
**Weight for length WFL**	−0.01 ± 1.85	−1.76 ± 0.95	−1.61 ± 0.88	−2.51 ± 1.68	−1.75 ± 0.99	−0.04 ± 0.96	0.08 ± 0.71	−0.10	0.01 ± 0.86	2.75 ± 1.68	2.13 ± 1.06	0.70	2.49 ± 1.50	<0.001	<0.001
<−3 SD	13 (3.0%)	8 (10.5%)	3 (5.6%)	2 (25.0%)	13 (9.4%)	0 (0%)	0 (0%)	0 (0%)	0 (0%)	0 (0%)	0 (0%)	0 (0%)	0 (0%)	<0.001	<0.001
≥−3 to − <−2 SD	41 (9.4%)	21 (27.6%)	15 (27.8%)	2 (25.0%)	38 (27.5%)	2 (1.7%)	0 (0%)	0 (0%)	2 (1.0%)	1 (1.8%)	0 (0%)	0 (0%)	1 (1.1%)		
≥−2 to <+1 SD	283 (64.8%)	46 (60.5%)	36 (66.7%)	4 (50.0%)	86 (62.3%)	103 (86.6%)	77 (89.5%)	1 (100%)	181 (87.9%)	9 (15.8%)	6 (17.1%)	1 (100%)	16 (17.2%)		
>+1 to ≤+2 SD	42 (9.6%)	1 (1.3%)	0 (0%)	0 (0%)	1 (0.7%)	10 (8.4%)	8 (9.3%)	0 (0%)	18 (8.7%)	11 (19.3%)	12 (34.3%)	0 (0%)	23 (24.7%)		
≥+2 − ≤+3 SD	26 (5.9%)	0 (0%)	0 (0%)	0 (0%)	0 (0%)	3 (2.5%)	1 (1.2%)	0 (0%)	4 (1.9%)	12 (21.1%)	10 (28.6%)	0 (0%)	22 (23.7%)		
>+3 SD	32 (7.3%)	0 (0%)	0 (0%)	0 (0%)	0 (0%)	1 (0.8%)	0 (0%)	0 (0%)	1 (0.5%)	24 (42.1%)	7 (20.0%)	0 (0%)	31 (33.3%)		
Total n = 437	437 (100%)	76 (100%)	54 (100%)	8 (100%)	138 (100%)	119 (100%)	86 (100%)	1 (100%)	206 (100%)	57 (100%)	35 (100%)	1 (100%)	93 (100%)		
**Weight for Age WFA**	−1.47 ± 1.42	−3.27 ± 0.93	−1.70 ± 0.75	0.29 ± 1.13	0.03 ± 0.63	−2.03 ± 0.80	0.60 ± 1.04	1.00	0.98 ± 0.79	0.60 ± 1.04	0.68 ± 0.69	1.86	0.50 ± 0.27	<0.001	<0.001
<−3 SD	60 (13.7%)	39 (51.3%)	3 (5.6%)	0 (0%)	42 (30.4%)	17 (14.3%)	0 (0%)	0 (0%)	17 (8.3%)	1 (1.8%)	0 (0%)	0 (0%)	1 (1.1%)	<0.001	<0.001
≥−3 to − ≥−2 SD	95 (21.7%)	34 (44.7%)	16 (29.6%)	1 (12.5%)	51 (37.0%)	41 (34.5%)	0 (0%)	0 (0%)	41 (19.9%)	3 (5.3%)	0 (0%)	0 (0%)	3 (3.2%)		
≥−1 SD	282 (64.5%)	3 (3.9%)	35 (64.8%)	7 (87.5%)	45 (32.6%)	61 (51.3%)	86 (100%)	1 (100%)	148 (71.8%)	53 (93.0%)	35 (100%)	1 (100%)	89 (95.7%)		
Total n = 437	437 (100%)	76 (100%)	54 (100%)	8 (100%)	138 (100%)	119 (100%)	86 (100%)	1 (100%)	206 (100%)	57 (100%)	35 (100%)	1 (100%)	93 (100%)		
**Height for Age HFA**	−2.18 ± 1.79	−3.25 ± 1.16	0.63 ± 1.00	3.51 ± 1.30	−1.83 ± 2.13	−3.19 ± 0.96	0.84 ± 0.81	2.66	−2.18 ± 1.50	−3.74 ± 1.23	−1.15 ± 0.75	2.02	−2.70 ± 1.72	<0.001	<0.001
<−3 SD	129 (29.5%)	35 (46.1%)	0 (0%)	0 (0%)	35 (25.4%)	55 (46.2%)	0 (0%)	0 (0%)	55 (26.7%)	40 (70.2%)	0 (0%)	0 (0%)	40 (43.0%)	<0.001	0.019
≥−3 to − ≥−2 SD	122 (27.9%)	41 (53.9%)	0 (0%)	0 (0%)	41 (29.7%)	64 (53.8%)	0 (0%)	0 (0%)	64 (31.1%)	17 (29.8%)	0 (0%)	0 (0%)	17 (18.3%)		
≥−1 SD	186 (42.6%)	0 (0%)	54 (100%)	8 (100%)	62 (44.9%)	0 (0%)	86 (100%)	1 (100%)	87 (42.2%)	0 (0%)	35 (100%)	1 (100%)	36 (38.7%)		
Total n = 437	437 (100%)	76 (100%)	54 (100%)	8 (100%)	138 (100%)	119 (100%)	86 (100%)	1 (100%)	206 (100%)	57 (100%)	35 (100%)	1 (100%)	93 (100%)		

MUAC, middle upper arm circumference. MAS, severe acute malnutrition. MAM, moderate acute malnutrition. ^1^ *p*-value was obtained and calculated using ANOVA or Chi-squared test for comparison between the nine Nutricode groups (1, 3, 5, 4, 6, 8, 7, 9, and 11). If any group had n = 0, it was excluded from the comparison. ^2^ *p*-value was obtained calculated using ANOVA or Chi-squared test for comparison between the three Nutricode categories (Thinness, Healthy Weight, and Overweight/Obesity). If any category had n = 0, it was excluded from the comparison. ^3^ Low birth weight was considered as <2.5 kg.

**Table 3:** Mother’s profile in relation to her child’s Nutricode.

		NUTRICODE													
	Total n = 437	Thinness (n = 138, 31.6%)				Healthy Weight (n = 206; 47.1%)				Overweight/Obesity (n = 93; 21.3%)				*p*-Value ^1^	*p*-Value ^2^
	Frequency (%) Mean ± SD	1 (n = 76; 17.4%)	3 (n = 54; 12.4%)	5 (n = 8; 1.8%)	Total (n = 138, 31.6%)	4 (n = 119; 47.1%)	6 (n = 86; 19.7%)	8 (n = 1; 0.2%)	Total (n = 206; 47.1%)	7 (n = 57; 13.0%)	9 (n = 35; 8.1%)	11 (n = 1; 0.2%)	Total (n = 93; 21.3%)		
**Age**	25.75 ± 6.16	25.86 ± 6.81	26.52 ± 6.70	26.00 ± 5.70	26.12 ± 6.67	26.08 ± 6.41	25.63 ± 5.55	22.00	25.87 ± 6.04	24.67 ± 6.02	25.46 ± 4.93	23.00	24.95 ± 5.59	0.892	0.343
<18	31 (7.1%)	7 (9.2%)	3 (5.6%)	0 (0%)	10 (7.2%)	9 (7.8%)	6 (7.0%)	1 (100%)	15 (7.3%)	4 (7.0%)	2 (5.7%)	0 (0%)	6 (6.5%)	0.991	0.692
18–29	297 (68.0%)	47 (61.8%)	37 (68.5%)	7 (87.5%)	91 (65.9%)	76 (63.9%)	59 (68.6%)	0 (0%)	136 (66.0%)	42 (73.7%)	27 (77.1%)	1 (100%)	70 (75.3%)		
30–39	90 (20.06%)	18 (23.7%)	10 (18.5%)	1 (12.5%)	29 (21.0%)	27 (22.7%)	19 (22.1%)	(0%)	46 (22.3%)	9 (15.8%)	6 (17.1%)	0 (0%)	15 (16.1%)		
40–49	19 (4.3%)	4 (5.3%)	4 (7.4%)	0 (0%)	8 (5.8%)	7 (5.9%)	2 (2.3%)	0 (0%)	9 (4.4%)	2 (3.5%)	0 (0%)	0 (0%)	2 (2.2%)		
Total	437 (100%)	76 (100%)	54 (100%)	8 (100%)	138 (100%)	119 (100%)	86 (100%)	1 (100%)	206 (100%)	57 (100%)	35 (100%)	1 (100%)	93 (100%)		
**Weight (kg)**	48.61 ± 8.04	45.60 ± 7.10	49.51 ± 10.05	50.60 ± 13.62	47.42 ± 8.96	47.06 ± 6.85	51.87 ± 8.60	38.30	49.03 ± 7.98	48.44 ± 5.89	50.80 ± 7.15	58.00	49.43 ± 6.49	<0.001	0.10
**Height (cm)**	152.65 ± 5.67	151.14 ± 5.20	154.37 ± 6.87	152.00 ± 5.90	152.46 ± 6.10	151.55 ± 5.44	154.30 ± 5.72	152.00	152.70 ± 5.69	152.96 ± 5.52	152.63 ± 3.99	153.00	152.84 ± 4.94	0.004	0.87
**BMI (kg/m^2^)**	20.77 ± 3.05	19.93 ± 2.88	20.58 ± 3.79	21.70 ± 4.91	20.28 ± 3.40	20.40 ± 2.69	21.66 ± 3.22	16.50	20.91 ± 2.99	20.73 ± 2.20	21.81 ± 2.79	24.80	21.18 ± 2.50	0.002	0.060
<18.5	108 (24.7%)	25 (32.9%)	21 (38.9%)	4 (50.0%)	50 (36.2%)	30 (25.2%)	15 (17.4%)	1 (100%)	46 (22.3%)	8 (14.0%)	4 (11.4%)	0 (0%)	12 (12.9%)	0.005	<0.001
18.5–24.9	289 (66.1%)	47 (61.8%)	25 (46.3%)	2 (25.0%)	74 (53.6%)	82 (68.9%)	59 (68.6%)	0 (0%)	141 (68.4%)	46 (80.7%)	27 (77.1%)	1 (100%)	74 (79.6%)		
25.0–29.9	34 (7.8%)	3 (3.9%)	5 (9.3%)	2 (25.0%)	10 (7.2%)	7 (5.9%)	10 (11.6%)	0 (0%)	17 (8.3%)	3 (5.3%)	4 (11.4%)	0 (0%)	7 (7.5%)		
>30	6 (1.4%)	1 (1.3%)	3 (5.6%)	0 (0%)	4 (2.9%)	0 (0%)	2 (2.3%)	0 (0%)	2 (1.0%)	0 (0%)	0 (0%)	0 (0%)	0 (0%)		
Total	437 (100%)	76 (100%)	54 (100%)	8 (100%)	138 (100%)	119 (100%)	86 (100%)	1 (100%)	206 (100%)	57 (100%)	35 (100%)	1 (100%)	93 (100%)		
**Parity**														0.773	0.164
Primiparous	161 (36.8%)	27 (35.5%)	22 (40.7%)	3 (37.5%)	52 (37.7%)	44 (37.0%)	27 (31.4%)	1 (100%)	72 (35.0%)	23 (40.4%)	14 (40.0%)	0 (0%)	37 (39.8%)		
2–3	203 (46.5%)	31 (40.8%)	21 (38.9%)	3 (37.5%)	55 (39.9%)	56 (47.1%)	48 (55.8%)	0 (0%)	104 (50.5%)	27 (47.4%)	16 (45.7%)	1 (100%)	44 (47.3%)		
≥4	73 (16.7%)	18 (23.7%)	11 (20.4%)	2 (25.9%)	31 (22.5%)	19 (16.0%)	11 (12.8%)	0 (0%)	30 (14.6%)	7 (12.3%)	5 (14.3%)	0 (0%)	12 (12.9%)		
Total	437 (100%)	76 (100%)	54 (100%)	8 (100%)	138 (100%)	119 (100%)	86 (100%)	1 (100%)	206 (100%)	57 (100%)	35 (100%)	1 (100%)	93 (100%)		
**Twin pregnancy ^3^**	13 (3%)	6 (7.9%)	1 (1.9%)	1 (12.5%)	8 (5.8%)	1 (0.8%)	2 (2.3%)	0 (0%)	3 (1.5%)	2 (3.5%)	0 (0%)	0 (0%)	2 (2.2%)	0.142	0.059
**Birth spacing < 24 months**	26 (5.9%)	6 (7.9%)	1 (1.9%)	0 (0%)	7 (5.1%)	12 (10.1%)	4 (4.7%)	0 (0%)	16 (7.8%)	2 (3.5%)	1 (2.9%)	0 (0%)	3 (3.2%)	0.451	0.018
**Education**														0.671	0.913
Illiterate	17 (3.9%)	4 (5.3%)	0 (0%)	1 (12.5%)	5 (3.6%)	4 (3.4%)	4 (4.7%)	0 (0%)	8 (3.9%)	4 (7.0%)	0 (0%)	0 (0%)	4 (4.3%)		
Primary	181 (41.4%)	40 (52.6%)	19 (35.2%)	3 (37.5%)	62 (44.9%)	50 (42.0%)	29 (33.7%)	0 (0%)	79 (38.3%)	26 (45.6%)	14 (40.0%)	0 (0%)	40 (43.0%)		
Secondary 1st cycle	193 (44.2%)	28 (36.8%)	27 (50.0%)	3 (37.5%)	58 (42.0%)	54 (45.4%)	42 (48.8%)	1 (100%)	97 (47.1%)	21 (36.8%)	16 (45.7%)	1 (100%)	38 (40.9%)		
Secondary 2nd cycle	46 (10.5%)	4 (5.3%)	8 (14.8%)	1 (12.5%)	13 (9.4%)	11 (9.2%)	11 (12.8%)	0 (0%)	22 (10.7%)	6 (10.5%)	5 (14.2%)	0 (0%)	11 (11.8%)		
Total	437 (100%)	76 (100%)	54 (100%)	8 (100%)	138 (100%)	119 (100%)	86 (100%)	1 (100%)	206 (100%)	57 (100%)	35 (100%)	1 (100%)	93 (100%)		
**Education level**														0.152	0.472
Primary or below	198 (45.3%)	44 (57.9%)	19 (35.2%)	4 (50.0%)	67 (48.6%)	54 (45.4%)	33 (38.4%)	0 (0%)	87 (42.2%)	30 (52.6%)	14 (40.0%)	0 (0%)	44 (47.3%)		
Secondary or above	239 (54.7%)	32 (42.1%)	35 (64.8%)	4 (50.0%)	71 (51.4%)	65 (54.6%)	53 (61.6%)	1 (100%)	119 (57.8%)	27 (47.4%)	21 (60.0%)	1 (100%)	49 (52.7%)		
**Occupation**														0.881	0.729
Farmer	335 (76.7%)	62 (81.6%)	39 (72.2%)	6 (75.0%)	107 (77.5%)	97 (81.5%)	60 (69.8%)	1 (100%)	158 (76.7%)	44 (77.2%)	25 (71.4%)	1 (100%)	70 (75.3%)		
Seller	41 (9.4%)	5 (6.6%)	5 (9.3%)	1 (12.5%)	11 (8.0%)	8 (6.7%)	11 (12.8%)	0 (0%)	19 (9.2%)	6 (10.5%)	5 (14.3%)	0 (0%)	11 (11.8%)		
Fisher	26 (5.9%)	4 (5.3%)	3 (5.6%)	0 (0%)	7 (5.1%)	7 (5.9%)	6 (7.0%)	0 (0%)	13 (6.3%)	4 (7.0%)	2 (5.7%)	0 (0%)	6 (6.5%)		
Housewife	5 (1.1%)	1 (1.3%)	0 (0%)	1 (12.5%)	2 (1.4%)	1 (0.8%)	1 (1.2%)	0 (0%)	2 (1.0%)	0 (0%)	1 (2.9%)	0 (0%)	1 (1.1%)		
Other	30 (6.9%)	4 (5.3%)	7 (13.0%)	0 (0%)	11 (8.0%)	6 (5.0%)	8 (9.3%)	0 (0%)	14 (6.8%)	3 (5.3%)	2 (5.7%)	0 (0%)	5 (5.4%)		
Total	437 (100%)	76 (100%)	54 (100%)	8 (100%)	138 (100%)	119 (100%)	86 (100%)	1 (100%)	206 (100%)	57 (100%)	35 (100%)	1 (100%)	93 (100%)		

^1^ *p*-value was obtained calculated using ANOVA or Chi-squared test for comparison between the nine Nutricode groups (1, 3, 5, 4, 6, 8, 7, 9, and 11). If any group had n = 0, it was excluded from the comparison. ^2^ *p*-value was obtained calculated using ANOVA or Chi-squared test for comparison between the three Nutricode categories (Thinness, Healthy Weight, and Overweight/Obesity). If any category had n = 0, it was excluded from the comparison. ^3^ Any twin pregnancy during lifetime.

**Table 4:** Maternal care and feeding practices in relation to her child’s Nutricode.

		NUTRICODE													
	Total n = 437	Thinness (n = 138, 31.6%)				Healthy Weight (n = 206; 47.1%)				Overweight/Obesity (n = 93; 21.3%)				*p*-Value ^1^	*p*-Value ^2^
	Frequency (%) Mean ± SD	1 (n = 76; 17.4%)	3 (n = 54; 12.4%)	5 (n = 8; 1.8%)	Total (n = 138, 31.6%)	4 (n = 119; 47.1%)	6 (n = 86; 19.7%)	8 (n = 1; 0.2%)	Total (n = 206; 47.1%)	7 (n = 57; 13.0%)	9 (n = 35; 8.1%)	11 (n = 1; 0.2%)	Total (n = 93; 21.3%)		
**ANC ^3^**														0.973	0.311
0	3 (0.7%)	0 (0%)	0 (0%)	0 (0%)	0 (0%)	2 (1.7%)	1 (1.2%)	0 (0%)	3 (1.5%)	0 (0%)	0 (0%)	0 (0%)	0 (0%)		
1	7 (1.6%)	2 (2.6%)	0 (0%)	0 (0%)	2 (1.4%)	2 (1.7%)	2 (2.3%)	0 (0%)	4 (1.9%)	1 (1.8%)	0 (0%)	0 (0%)	1 (1.1%)		
2–3	31 (7.1%)	10 (13.2%)	3 (5.6%)	1 (12.5%)	14 (10.1%)	6 (5%)	4 (4.7%)	0 (0%)	10 (4.9%)	5 (8.8%)	2 (5.7%)	0 (0%)	7 (7.5%)		
≥4	396 (90.6%)	64 (84.2%)	51 (94.4%)	7 (87.5%)	122 (88.4%)	109 (91.6%)	79 (91.9%)	1 (100%)	189 (91.7%)	51 (89.5%)	33 (94.3%)	1 (100%)	85 (91.4%)		
Total	437 (100%)	76 (100%)	54 (100%)	8 (100%)	138 (100%)	119 (100%)	86 (100%)	1 (100%)	206 (100%)	57 (100%)	35 (100%)	1 (100%)	93 (100%)		
**IFA ^4^ supplementation**	366 (83.8%)	59 (77.6%)	45 (83.3%)	7 (87.5%)	111 (80.4%)	101 (84.9%)	73 (84.9%)	0 (0%)	174 (84.5%)	50 (87.7%)	30 (85.7%)	1 (100%)	81 (87.1%)	0.396	0.374
**Type of delivery**														<0.001	0.138
Vaginal	402 (92.0%)	74 (97.4%)	46 (85.2%)	6 (75.0%)	126 (91.3%)	112 (94.1%)	78 (90.7%)	0 (0%)	190 (92.2%)	54 (94.7%)	32 (91.4%)	0 (0%)	86 (95.2%)		
Operative vaginal birth	23 (5.3%)	0 (0%)	4 (7.4%)	1 (12.5%)	5 (3.6%)	6 (5.0%)	7 (8.1%)	1 (100%)	14 (6.8%)	1 (1.8%)	3 (8.6%)	0 (0%)	4 (4.3%)		
Caesarean section	12 (2.7%)	2 (2.6%)	4 (7.4%)	1 (12.5%)	7 (5.1%)	1 (0.8%)	1 (1.2%)	0 (0%)	2 (1.0%)	2 (3.5%)	0 (0%)	1 (100%)	3 (3.2%)		
Total	437 (100%)	76 (100%)	54 (100%)	8 (100%)	138 (100%)	119 (100%)	86 (100%)	1 (100%)	206 (100%)	57 (100%)	35 (100%)	1 (100%)	93 (100%)		
**Place of delivery**														0.267	0.082
Home	157 (35.9%)	29 (38.2%)	16 (29.6%)	2 (25%)	47 (34.1%)	53 (44.5%)	30 (34.9%)	1 (100%)	84 (40.8%)	16 (28.1%)	10 (28.6%)	0 (0%)	26 (28.0%)		
Health centre	280 (64.1%)	47 (61.8%)	38 (70.4%)	6 (75.0%)	91 (65.9%)	66 (55.5%)	56 (65.1%)	0 (0%)	122 (59.2%)	41 (71.9%)	25 (71.4%)	1 (100%)	67 (72.0%)		
Total	437 (100%)	76 (100%)	54 (100%)	8 (100%)	138 (100%)	119 (100%)	86 (100%)	1 (100%)	206 (100%)	57 (100%)	35 (100%)	1 (100%)	93 (100%)		
**Reason in case of home delivery**														0.801	0.573
Upcoming birth	72 (45.0%)	15 (51.7%)	6 (37.5%)	2 (100%)	23 (48.9%)	28 (50.9%)	10 (32.3%)	1 (100%)	39 (44.9%)	5 (31.3%)	5 (50.0%)	-	10 (38.5%)		
Personal choice	60 (37.5%)	7 (24.1%)	5 (31.3%)	0 (0%)	12 (25.5%)	20 (36.4%)	17 (54.8%)	0 (0%)	37 (42.5%)	6 (37.5%)	5 (50.0%)	-	11 (42.3%)		
Transport issues	19 (11.9%)	5 (17.2%)	4 (25.0%)	0 (0%)	9 (19.1%)	4 (7.3%)	3 (9.7%)	0 (0%)	7 (8.0%)	3 (18.8%)	0 (0%)	-	3 (11.5%)		
Homecare for a matron	4 (2.5%)	0 (0%)	1 (6.3%)	0 (0%)	1 (2.1%)	2 (3.6%)	0 (0%)	0 (0%)	2 (2.3%)	1 (6.3%)	0 (0%)	-	1 (3.8%)		
Lack of money	4 (2.5%)	2 (6.9%)	0 (0%)	0 (0%)	2 (4.3%)	0 (0%)	1 (3.2%)	0 (0%)	1 (1.1%)	1 (6.3%)	0 (0%)	-	1 (3.8%)		
Absence of health staff	1 (0.6%)	0 (0%)	0 (0%)	0 (0%)	0 (0%)	1 (1.8%)	0 (0%)	0 (0%)	1 (1.1%)	0 (0%)	0 (0%)	-	0 (0%)		
**Early breastfeeding initiation ^5^**	251 (57.4%)	34 (44.7%)	29 (53.7%)	5 (62.5%)	68 (49.3%)	70 (58.8%)	50 (58.1%)	0 (0%)	120 (58.3%)	37 (64.9%)	25 (71.4%)	1 (100%)	63 (67.6%)	0.162	0.021
**EBF ^6^** (n = 385)	170 (44.2%)	26 (36.6%)	25 (51.0%)	4 (50.0%)	55 (43.0%)	50 (43.9%)	32 (46.4%)	0 (0%)	82 (44.6%)	23 (48.9%)	10 (40.0%)	0 (0%)	33 (45.2%)	0.761	0.949
**Breastfeeding up to 1-year** (n = 304)	292 (96.1%)	64 (95.5%)	35 (100%)	7 (100%)	106 (97.2%)	89 (91.8%)	45 (97.8%)	1 (100%)	135 (93.8%)	34 (100%)	16 (100%)	1 (100%)	51 (100%)	0.346	0.103
**Early weaning ^7^** (n = 368)	224 (60.9%)	44 (62.9%)	26 (55.3%)	5 (62.5%)	75 (60.0%)	70 (62.5%)	36 (58.1%)	0 (0%)	106 (60.6%)	29 (65.9%)	14 (60.9%)	0 (0%)	43 (63.2%)	0.792	0.901
**Improved mother’s diet during pregnancy**	235 (53.8%)	30 (39.5%)	30 (55.6%)	4 (50.0%)	64 (46.4%)	68 (57.1%)	45 (52.3%)	1 (100%)	114 (55.3%)	33 (57.9%)	23 (65.7%)	1 (100%)	57 (61.3%)	0.195	0.064
**Improved mother’s diet during breastfeeding**	182 (41.6%)	25 (32.9%)	20 (37.0%)	2 (25.0%)	47 (34.1%)	56 (47.1%)	37 (43.0%)	1 (100%)	94 (45.6%)	22 (38.6%)	18 (51.4%)	1 (100%)	41 (44.1%)	0.283	0.093
**Iodized salt**	330 (75.5%)	54 (71.1%)	39 (72.2%)	7 (87.5%)	100 (74.7%)	95 (79.8%)	61 (70.9%)	1 (100%)	157 (76.2%)	42 (73.7%)	30 (85.7%)	1 (100%)	73 (78.5%)	0.576	0.551

^1^ *p*-value was obtained calculated using ANOVA or Chi-squared test for comparison between the nine Nutricode groups (1, 3, 5, 4, 6, 8, 7, 9, and 11). If any group had n = 0, it was excluded from the comparison. ^2^ *p*-value was obtained calculated using ANOVA or Chi-squared test for comparison between the three Nutricode categories (Thinness, Healthy Weight, and Overweight/Obesity). If any category had n = 0, it was excluded from the comparison. ^3^ ANC: antenatal care. ^4^ IFA: iron and folic acid supplementation during last pregnancy. ^5^ Early breastfeeding initiation within 1 h after birth. ^6^ Exclusive breastfeeding for the first 6 months. ^7^ Early weaning before 6 months.

**Table 5:** Socioeconomic information about the household.

		NUTRICODE													
	Total n = 437	Thinness (n = 138, 31.6%)				Healthy Weight (n = 206; 47.1%)				Overweight/Obesity (n = 93; 21.3%)				*p*-Value ^1^	*p*-Value ^2^
	Frequency (%) Mean ± SD	1 (n = 76; 17.4%)	3 (n = 54; 12.4%)	5 (n = 8; 1.8%)	Total (n = 138, 31.6%)	4 (n = 119; 47.1%)	6 (n = 86; 19.7%)	8 (n = 1; 0.2%)	Total (n = 206; 47.1%)	7 (n = 57; 13.0%)	9 (n = 35; 8.1%)	11 (n = 1; 0.2%)	Total (n = 93; 21.3%)		
**Family size**	4.59 ± 1.69	4.59 ± 1.52	4.65 ± 1.68	4.88 ± 1.13	4.63 ± 1.56	4.56 ± 1.80	4.66 ± 1.51	3.00	4.60 ± 1.68	4.67 ± 2.02	4.31 ± 1.67	5.00	4.54 ± 1.89	0.962	0.921
**Dimension of the house**	28.81 ± 24.12	26.21 ± 18.54	30.58 ± 23.25	26.62 ± 14.80	27.95 ± 20.32	30.44 ± 28.16	32.53 ± 31.06	16.00	31.24 ± 29.30	23.46 ± 13.91	26.85 ± 14.68	20.00	24.70 ± 14.16	0.515	0.086
**Overcrowded housing ^3^ (m^2^/person)**	211 (48.3%)	43 (56.6%)	22 (40.7%)	4 (50.0%)	69 (50.0%)	57 (47.9%)	39 (45.3%)	0 (0%)	96 (46.6%)	30 (52.6%)	15 (42.9%)	1 (100%)	46 (49.5%)	0.599	0.202
**Low–middle income ^4^**	328 (75.1%)	62 (81.6%)	43 (79.6%)	3 (37.5%)	108 (78.3%)	92 (77.3%)	59 (68.6%)	1 (100%)	152 (73.8%)	44 (77.2%)	24 (68.6%)	0 (0%)	68 (73.1%)	0.061	0.576
**Source of drinking water**														0.884	0.414
Public standpipe	149 (34.1%)	23 (30.3%)	14 (25.9%)	2 (25.0%)	39 (28.3%)	47 (39.5%)	29 (33.7%)	0 (0%)	76 (36.9%)	23 (40.4%)	10 (28.6%)	1 (100%)	34 (36.6%)		
Protected well	285 (65.2%)	52 (68.4%)	40 (74.1%)	6 (75.0%)	98 (71.0%)	71 (59.7%)	34 (59.6%)	1 (100%)	128 (62.1%)	34 (59.6%)	25 (71.4%)	0 (0%)	59 (63.4%)		
Unprotected spring	3 (0.7%)	1 (1.3%)	0 (0%)	0 (0%)	1 (0.7%)	1 (0.8%)	1 (0.8%)	0 (0%)	2 (1.0%)	0 (0%)	0 (0%)	0 (0%)	0 (0%)		
**Rice < 6 months**	140 (32.0%)	27 (35.5%)	12 (22.2%)	4 (50%)	43 (31.2%)	36 (30.3%)	22 (25.6%)	1 (100%)	59 (28.6%)	24 (42.1%)	14 (40.0%)	1 (100%)	38 (40.9%)	0.153	0.114
**Toilet facility**	398 (91.1%)	66 (86.8%)	51 (94.4%)	8 (100%)	125 (90.6%)	106 (89.1%)	82 (95.3%)	1 (100%)	189 (91.7%)	51 (89.5%)	1 (100%)	1 (100%)	84 (90.3%)	0.631	0.893
**Walking distance from water (min)**	9.29 ± 11.25	13.33 ± 15.94	7.65 ± 8.17	5.50 ± 4.81	10.65 ± 13.24	9.00 ± 9.83	9.19 ± 10.25	15.00	9.11 ± 9.96	7.72 ± 10.14	7.69 ± 11.50	3.00	7.66 ± 10.57	0.079	0.134
**Walking distance from HC ^5^ (min)**	47.67 ± 33.04	49.41 ± 42.67	42.67 ± 32.34	39.25 ± 27.60	46.18 ±33.02	52.26 ± 34.98	48.81 ± 36.54	30.00	50.71 ±35.53	42.90 ± 26.97	42.17 ± 24.49	90.00	43.13 ± 26.24	0.396	0.151
**Transport availability**	113 (25.9%)	22 (28.9%)	14 (25.9%)	3 (37.5%)	39 (28.3%)	26 (21.8%)	25 (29.1%)	1 (100%)	52 (25.2%)	10 (17.5%)	11 (31.4%)	1 (100%)	22 (23.7%)	0.223	0.709
**Land’s owner**	226 (51.7%)	34 (44.7%)	30 (55.6%)	4 (50.0%)	62 (44.9%)	70 (58.8%)	46 (53.5%)	1 (100%)	116 (56.3%)	28 (49.1%)	19 (54.3%)	1 (100%)	48 (51.6%)	0.491	0.122

^1^ *p*-value was obtained calculated using ANOVA or Chi-squared test for comparison between the nine Nutricode groups (1, 3, 5, 4, 6, 8, 7, 9, and 11). If any group had n = 0, it was excluded from the comparison. ^2^ *p*-value was obtained calculated using ANOVA or Chi-squared test for comparison between the three Nutricode categories (Thinness, Healthy Weight, and Overweight/Obesity). If any category had n = 0, it was excluded from the comparison. ^3^ Overcrowding rate was considered as <5 m^2^ of floor area per person. ^4^ Low middle income was established as <MGA Ar 200,000 (national currency of Madagascar Ar). ^5^ HC: health centre.

**Table 6:** Dietary diversity of the mothers in relation to their children’s Nutricode.

		NUTRICODE													
	Total n = 437	Thinness (n = 138, 31.6%)				Healthy Weight (n = 206; 47.1%)				Overweight/Obesity (n = 93; 21.3%)				*p*-Value ^1^	*p*-Value ^2^
	Frequency (%) Mean ± SD	1 (n = 76; 17.4%)	3 (n = 54; 12.4%)	5 (n = 8; 1.8%)	Total (n = 138, 31.6%)	4 (n = 119; 47.1%)	6 (n = 86; 19.7%)	8 (n = 1; 0.2%)	Total (n = 206; 47.1%)	7 (n = 57; 13.0%)	9 (n = 35; 8.1%)	11 (n = 1; 0.2%)	Total (n = 93; 21.3%)		
**<5 groups**	144 (33%)	30 (39.5%)	17 (31.5%)	3 (37.5%)	50 (36.2%)	39 (32.8%)	31 (36%)	0 (0%)	70 (34.0%)	12 (21.1%)	12 (34.3%)	0 (0%)	24 (25.8%)	0.577	0.265
**Grains, white roots and tubers.**	437 (100%)	76 (100%)	54 (100%)	8 (100%)	138 (100%)	119 (100%)	86 (100%)	1 (100%)	206 (100%)	57 (100%)	35 (100%)	1 (100%)	93 (100%)	1.0	1.0
**Pulses**	150 (34.3%)	31 (40.8%)	18 (33.3%)	4 (50.0%)	53 (38.4%)	46 (38.7%)	26 (30.2%)	0 (0%)	72 (35.0%)	18 (31.6%)	7 (20.0%)	0 (0%)	26 (26.9%)	0.40	0.19
**Nuts and seeds**	86 (19.7%)	14 (18.4%)	8 (14.8%)	1 (12.5%)	23 (16.7%)	31 (26.1%)	13 (15.1%)	0 (0%)	44 (21.4%)	13 (22.8%)	5 (14.3%)	1 (100%)	19 (20.4%)	0.22	0.55
**Dairy products**	128 (29.3%)	23 (30.3%)	18 (33.3%)	4 (50.0%)	45 (32.6%)	35 (29.4%)	24 (27.9%)	0 (0%)	59 (28.6%)	14 (24.6%)	9 (25.7%)	1 (100%)	24 (25.8%)	0.66	0.52
**Meats, poultry, fish**	301 (68.9%)	45 (59.2%)	41 (75.9%)	5 (62.5%)	91 (65.9%)	75 (63.0%)	63 (73.3%)	0 (0%)	138 (67.0%)	43 (75.4%)	28 (80.0%)	1 (100%)	72 (77.4%)	0.10	0.13
**Eggs**	44 (10.1%)	5 (6.6%)	4 (7.4%)	1 (12.5%)	10 (7.2%)	12 (10.1%)	11 (12.8%)	0 (0%)	23 (11.2%)	7 (12.3%)	4 (11.4%)	0 (0%)	11 (11.8%)	0.95	0.40
**Dark green leafy vegetables**	322 (73.7%)	54 (71.1%)	37 (68.5%)	7 (87.5%)	98 (71.0%)	94 (79.0%)	60 (69.8%)	1 (100%)	155 (75.2%)	44 (77.2%)	24 (68.6%)	1 (100%)	69 (74.2%)	0.67	0.68
**Other Vitamin A rich fruits and vegetables**	250 (57.2%)	43 (56.6%)	33 (61.1%)	3 (37.5%)	79 (57.2%)	69 (58.0%)	44 (51.2%)	1 (100%)	114 (55.3%)	35 (61.4%)	21 (60.0%)	1 (100%)	57 (61.3%)	0.76	0.63
**Other vegetables**	328 (75.1%)	53 (69.7%)	37 (68.5%)	5 (62.5%)	95 (68.8%)	92 (77.3%)	67 (77.9%)	1 (100%)	160 (77.7%)	44 (77.2%)	28 (80.0%)	1 (100%)	73 (78.5%)	0.76	0.12
**Other fruits**	230 (52.5%)	39 (51.3%)	28 (51.9%)	6 (75.0%)	73 (52.9%)	63 (52.9%)	45 (52.3%)	1 (100%)	109 (52.9%)	32 (56.1%)	16 (45.7%)	0 (0%)	48 (51.6%)	0.79	0.97

^1^ *p*-value was obtained calculated using ANOVA or Chi-squared test for comparison between the nine Nutricode groups (1, 3, 5, 4, 6, 8, 7, 9, and 11). If any group had n = 0, it was excluded from the comparison. ^2^ *p*-value was obtained calculated using ANOVA or Chi-squared test for comparison between the three Nutricode categories (Thinness, Healthy Weight, and Overweight/Obesity). If any category had n = 0, it was excluded from the comparison.

## Data Availability

The datasets presented in this article are not readily available due to ethical personal data sharing restrictions. Requests to access the datasets should be directed to the corresponding author.

## References

[1] UNICEF, World Health Organization (2023). World Bank Group Levels and Trends in Child Malnutrition Child Malnutrition: UNICEF/WHO/World Bank Group Joint Child Malnutrition Estimates: Key Findings of the 2023 Edition.

[2] Javaid A., Syed S. (2022). Infant Nutrition in Low-and Middle-Income Countries. Clin. Perinatol..

[3] Moradi S., Mirzababaei A., Mohammadi H., Moosavian S.P., Arab A., Jannat B., Mirzaei K. (2019). Food insecurity and the risk of undernutrition complications among children and adolescents: A systematic review and meta-analysis. Nutrition.

[4] Kosaka S., Umezaki M. (2017). A systematic review of the prevalence and predictors of the double burden of malnutrition within households. Br. J. Nutr..

[5] Popkin B.M., Corvalan C., Grummer-Strawn L.M. (2020). Dynamics of the double burden of malnutrition and the changing nutrition reality. Lancet.

[6] Santana dos Santos I.K., Borges dos Santos Pereira D., Cumpian Silva J., de Oliveira Gallo C., de Oliveira M.H., Pereira de Vasconcelos L.C., Conde W.L. (2024). Frequency of anthropometric implausible values estimated from different methodologies: A systematic review and meta-analysis. Nutr. Rev..

[7] Hron B.M., Duggan C.P. (2020). Pediatric undernutrition defined by body composition—Are we there yet?. Am. J. Clin. Nutr..

[8] Mwala N.N., Borkent J.W., van der Meij B.S., de van der Schueren M.A. (2025). Challenges in identifying malnutrition in obesity; An overview of the state of the art and directions for future research. Nutr. Res. Rev..

[9] Dewan D., Gupta R., Kumar D. (2015). Can we rely solely on conventional measures to estimate undernutrition among under-fives?. Indian J. Community Health.

[10] Sen J., Dey S., Mondal N. (2011). Conventional nutritional indices and Composite Index of Anthropometric Failure: Which seems more appropriate for assessing under-nutrition among children? A cross-sectional study among school children of the Bengalee Muslim Population of North Bengal, Indi. Ital. J. Public Health.

[11] Anwar F., Gupta M.K., Prabha C., Srivastava R.K. (2013). Malnutrition among rural Indian children: An assessment using web of indices. Int. J. Public Health Epidemiol..

[12] Nandy S., Irving M., Gordon D., Subramanian S.V., Smith G.D. (2005). Poverty, child undernutrition and morbidity: New evidence from India. Bull. World Health Organ..

[13] Green Corkins K., Teague E.E. (2017). Pediatric nutrition assessment: Anthropometrics to zinc. Nutr. Clin. Pract..

[14] Teshome E.M., Kiprotich P., Andango P.E. (2017). Paradigm shift: Efficient and cost effective real-time nutritional assessment technique. Afr. J. Food Agric. Nutr. Dev..

[15] Trehan I. (2020). Anthropometry’s promise and pitfalls in the diagnosis of undernutrition among infants. Arch. Dis. Child..

[16] Raiten D.J., Steiber A.L., Dary O., Bremer A.A. (2024). The Value of an Ecological Approach to Improve the Precision of Nutritional Assessment: Addressing Contributors and Implications of the “Multiple Burdens of Malnutrition”. Nutrients.

[17] Frison S., Kerac M., Checchi F., Prudhon C. (2016). Anthropometric indices and measures to assess change in the nutritional status of a population: A systematic literature review. BMC Nutr..

[18] Food & Agriculture Organization, International Fund for Agricultural Development, UNICEF, World Food Programme, World Health Organization (2023). The State of Food Security and Nutrition in the World 2023: Urbanization, Agrifood Systems Transformation and Healthy Diets across the Rural–Urban Continuum.

[19] Roseboom T.J. (2019). Epidemiological evidence for the developmental origins of health and disease: Effects of prenatal undernutrition in humans. J. Endocrinol..

[20] Grey K., Gonzales G.B., Abera M., Lelijveld N., Thompson D., Berhane M., Abdissa A., Girma T., Kerac M. (2021). Severe malnutrition or famine exposure in childhood and cardiometabolic non-communicable disease later in life: A systematic review. BMJ Glob. Health.

[21] Briend A., Berkley J.A. (2016). Long term health status of children recovering from severe acute malnutrition. Lancet Glob. Health.

[22] Prasadajudio M., Devaera Y., Noormanto N., Kuswiyanto R.B., Sudarmanto B., Andriastuti M., Sidiartha I.G.L., Sitorus N.L., Basrowi R.W. (2023). Disease-related malnutrition in pediatric patients with chronic disease: A developing country perspective. Curr. Dev. Nutr..

[23] Mwene-Batu P., Bisimwa G., Ngaboyeka G., Dramaix M., Macq J., Hermans M.P., Lemogoum D., Donnen P. (2021). Severe acute malnutrition in childhood, chronic diseases, and human capital in adulthood in the Democratic Republic of Congo: The Lwiro Cohort Study. Am. J. Clin. Nutr..

[24] Nguyen L.T., Pollock C.A., Saad S. (2023). Nutrition and developmental origins of kidney disease. Nutrients.

[25] Ahmed S., PrayGod G., Lee N.R., Kelly P., Trilok-Kumar G., Chisenga M., Kweka B., Faurholt-Jepsen D., Krogh-Madsen R., Shaw J.A. (2022). Long-term health after Severe Acute Malnutrition in children and adults-the role of the Pancreas (SAMPA): Protocol. F1000Research.

[26] Cunningham-Rundles S., McNeeley D.F., Moon A. (2005). Mechanisms of nutrient modulation of the immune response. J. Allergy Clin. Immunol..

[27] Martorell R. (1999). The nature of child malnutrition and its long-term implications. Food Nutr. Bull..

[28] Bloem M. (2007). The 2006 WHO child growth standards. BMJ.

[29] World Bank Group (2006). Repositioning Nutrition as Central to Development: A Strategy for Large Scale Action.

[30] Martins P.A., Hoffman D.J., Fernandes M.T.B., Nascimento C.R., Roberts S.B., Sesso R., Sawaya A.L. (2004). Stunted children gain less lean body mass and more fat mass than their non-stunted counterparts: A prospective study. Br. J. Nutr..

[31] Florêncio T.M., Bueno N.B., Clemente A.P., Albuquerque F.C., Britto R.P., Ferriolli E., Sawaya A.L. (2015). Weight gain and reduced energy expenditure in low-income Brazilian women living in slums: A 4-year follow-up study. Br. J. Nutr..

[32] Bose A., Mondal N., Sen J. (2022). Household levels of double burden of malnutrition in low–middle-income countries: A review. J. Anthropol. Surv. India.

[33] Kolčić I. (2012). Double burden of malnutrition: A silent driver of double burden of disease in low–and middle–income countries. J. Glob. Health.

[34] Tzioumis E., Kay M.C., Bentley M.E., Adair L.S. (2016). Prevalence and trends in the childhood dual burden of malnutrition in low-and middle-income countries, 1990–2012. Public Health Nutr..

[35] Caleyachetty R., Thomas G.N., Kengne A.P., Echouffo-Tcheugui J.B., Schilsky S., Khodabocus J., Uauy R. (2018). The double burden of malnutrition among adolescents: Analysis of data from the Global School-Based Student Health and Health Behavior in School-Aged Children surveys in 57 low-and middle-income countries. Am. J. Clin. Nutr..

[36] Patel S.K., LBD Double Burden of Malnutrition Collaborators (2020). Mapping local patterns of childhood overweight and wasting in low-and middle-income countries between 2000 and 2017. Nat. Med..

[37] Seferidi P., Hone T., Duran A.C., Bernabe-Ortiz A., Millett C. (2022). Global inequalities in the double burden of malnutrition and associations with globalisation: A multilevel analysis of Demographic and Health Surveys from 55 low-income and middle-income countries, 1992–2018. Lancet Glob. Health.

[38] Were J.M., Stranges S., Sharma I., Vargas-Gonzalez J.C., Campbell M.K. (2022). Abstract P101: The Double Burden of Malnutrition among Women and Preschool Children in Low-and Middle-income Countries: A Scoping Review and Thematic Analysis of The Literature. Circulation.

[39] Wrottesley S.V., Lamper C., Pisa P.T. (2016). Review of the importance of nutrition during the first 1000 days: Maternal nutritional status and its associations with fetal growth and birth, neonatal and infant outcomes among African women. J. Dev. Orig. Health Dis..

[40] Adu-Afarwuah S., Lartey A., Dewey K.G. (2017). Meeting nutritional needs in the first 1000 days: A place for small-quantity lipid-based nutrient supplements. Ann. N. Y. Acad. Sci..

[41] Fauziah L., Purnasari H., Riana A., Ardayani T. (2023). 1000 First Days of Life As An Effort to Prevention and Prevention Stunting in Rw 08 North Margahayu. Aktual J. Pengabdi. Kpd. Masy..

[42] Climate-Affected Madagascar Adapts to New Reality: A UN Resident Coordinator Blog. https://news.un.org/en/story/2024/02/1146737.

[43] The Burden on Those Least Responsible: The Impact of Climate Change on Maternal Health in Madagascar. https://madagascar.co.uk/blog/2025/04/burden-those-least-responsible-impact-climate-change-maternal-health-madagascar.

[44] Selem-Solís J.E., Alcocer-Gamboa A., Hattori-Hara M., Esteve-Lanao J., Larumbe-Zabala E. (2018). Nutrimetry: BMI assessment as a function of development. Endocrinol. Diabetes Nutr..

[45] (2014). General Assembly of the World Medical Association World Medical Association Declaration of Helsinki: Ethical principles for medical research involving human subjects. J. Am. Coll. Dent..

[46] McIntosh N., Bates P., Brykczynska G., Dunstan G., Goldman A., Harvey D., Larcher V., McCrae D., McKinnon A., Patton M. (2000). Guidelines for the ethical conduct of medical research involving children. Royal College of Paediatrics, Child Health: Ethics Advisory Committee. Arch. Dis. Child..

[47] Rotella R., Soriano J.M., Peraita-Costa I., Llopis-González A., Morales-Suarez-Varela M. (2024). Evaluation of nutritional status using the minimum dietary diversity for women of reproductive age (MDD-W) tool in breastfeeding mothers in Madagascar. Trop. Med. Int. Health.

[48] Minimum Dietary Diversity for Women: A Guide for Measurement. http://www.fao.org/3/a-i5486e.pdf.

[49] World Health Organization (2010). WHO Anthro for Personal Computers.

[50] Arsenault J.E., Mora-Plazas M., Forero Y., Lopez-Arana S., Jáuregui G., Baylin A., Gordon P.M., Villamor E. (2011). Micronutrient and anthropometric status indicators are associated with physical fitness in Colombian schoolchildren. Br. J. Nutr..

[51] Poh B.K., Ng B.K., Haslinda M.D.S., Shanita S.N., Wong J.E., Budin S.B., Ng L.O., Khouw I., Norimah A.K. (2013). Nutritional status and dietary intakes of children aged 6 months to 12 years: Findings of the Nutrition Survey of Malaysian Children (SEANUTS Malaysia). Br. J. Nutr..

[52] Tapia-Veloz E., Guillén M., Trelis M., Carpio-Arias T.V., Gozalbo M. (2023). Assessment of the health status of Spanish schoolchildren based on nutrimetry, lifestyle and intestinal parasites. Nutrients.

[53] Tapia-Veloz E., Gozalbo M., Tapia-Veloz G., Carpio-Arias T.V., Trelis M., Guillén M. (2022). Evaluation of school children nutritional status in Ecuador using nutrimetry: A proposal of an education protocol to address the determinants of malnutrition. Nutrients.

[54] Solís J.E.S., Gamboa A.A., Hara M.H. (2018). Nutrimetría de z-IMC vs z-peso en función al desarrollo lineal en edades de 0 a 30 meses. Rev. Esp. Nutr. Comunitaria = Span. J. Community Nutr..

[55] Selem-Solís J.E., Richaud-Lara M., Larumbe-Zabala E., Esteve-Lanao J., Alcocer-Gamboa A. Nutrimetry: The scoring of Height and BMI. Proceedings of the III World Congress of Public Health Nutrition.

[56] Alfano M.V., Gozalbo M., Tapia-Veloz G., Guirao V., Soriano J.M., Trelis M. (2025). Nutrimetry and Evaluation of Intestinal Parasites and Anaemia in Malnourished Schoolchildren from Toliara (Madagascar). Children.

[57] Morales-Suárez-Varela M., Peraita-Costa I., Llopis-Morales A., Llopis-González A. (2024). Cross-Sectional Assessment of Nutritional Status, Dietary Intake, and Physical Activity Levels in Children (6–9 Years) in Valencia (Spain) Using Nutrimetry. Nutrients.

[58] Victora C.G., Adair L., Fall C., Hallal P.C., Martorell R., Richter L., Sachdev H.S. (2008). Maternal and child undernutrition: Consequences for adult health and human capital. Lancet.

[59] Mason J.B., Shrimpton R., Saldanha L.S., Ramakrishnan U., Victora C.G., Girard A.W., McFarland D.A., Martorell R. (2014). The first 500 days of life: Policies to support maternal nutrition. Glob. Health Action.

[60] Pileggi V.N., Oladapo O.T., de Souza H.C.C., Castro C.P., Abraham A.O., Akintan A.L., Idris H.A., Oyeneyin L.O., Souza J.P., Camelo J.S. (2020). Maternal BMI at the time of birth and selected risk factors associated with severe neonatal outcomes: A secondary analysis of the WHO Better Outcomes in Labour Difficulty (BOLD) project. Br. J. Nutr..

[61] Walle B.M., Adekunle A.O., Arowojolu A.O., Dugul T.T., Mebiratie A.L. (2020). Micronutrients deficiency and their associations with pregnancy outcomes: A review. Nutr. Diet. Suppl..

[62] Yang W., Han N., Jiao M., Chang X., Liu J., Zhou Q., Wang H. (2023). Maternal diet quality during pregnancy and its influence on low birth weight and small for gestational age: A birth cohort in Beijing, China. Br. J. Nutr..

[63] Kelleher C.C., Viljoen K., Khalil H., Somerville R., O’Brien J., Shrivastava A., Murrin C. (2014). Longitudinal follow-up of the relationship between dietary intake and growth and development in the Lifeways cross-generation cohort study 2001–2013. Proc. Nutr. Soc..

[64] Kozuki N., Lee A.C., Katz J. (2012). Child Health Epidemiology Reference Group Moderate to severe, but not mild, maternal anemia is associated with increased risk of small-for-gestational-age outcomes 3. J. Nutr..

[65] Epiu I., Byamugisha J., Kwikiriza A., Autry M.A. (2018). Health and sustainable development; strengthening peri-operative care in low income countries to improve maternal and neonatal outcomes. Reprod. Health.

[66] Blencowe H., Cousens S., Jassir F.B., Say L., Chou D., Mathers C., Hogan D., Shiekh S., Qureshi Z.U., You D. (2016). National, regional, and worldwide estimates of stillbirth rates in 2015, with trends from 2000: A systematic analysis. Lancet Glob. Health.

[67] World Health Organization (2013). Guideline: Updates on the Management of Severe Acute Malnutrition in Infants and Children.

[68] Golden C.D., Rice B.L., Randriamady H.J., Vonona A.M., Randrianasolo J.F., Tafangy A.N., Andrianantenaina M.Y., Arisco N.J., Emile G.N., Lainandrasana F. (2020). Study protocol: A cross-sectional examination of socio-demographic and ecological determinants of nutrition and disease across Madagascar. Front. Public Health.

[69] Matekenya D., Mulangu F., Newhouse D. (2025). Malnourished but not destitute: The spatial interplay between nutrition and poverty in Madagascar. J. Int. Dev..

[70] Shrimpton R., Rokx C. (2012). The Double Burden of Malnutrition. A Review of Global Evidence.

[71] Smith L.C., Haddad L. (2015). Reducing child undernutrition: Past drivers and priorities for the post-MDG era. World Dev..

[72] Darmon N., Drewnowski A. (2008). Does social class predict diet quality?. Am. J. Clin. Nutr..

[73] Giskes K., Avendaňo M., Brug J., Kunst A.E. (2010). A systematic review of studies on socioeconomic inequalities in dietary intakes associated with weight gain and overweight/obesity conducted among European adults. Obes. Rev..

[74] Chase C., Ngure F. (2016). Multisectoral Approaches to Improving Nutrition: Water, Sanitation, and Hygiene.

[75] Arimond M., Ruel M.T. (2004). Dietary diversity is associated with child nutritional status: Evidence from 11 demographic and health surveys. J. Nutr..

[76] Dror D.K., Allen L.H. (2011). The importance of milk and other animal-source foods for children in low-income countries. Food Nutr. Bull..

[77] Girard A.W., Self J.L., McAuliffe C., Olude O. (2012). The effects of household food production strategies on the health and nutrition outcomes of women and young children: A systematic review. Paediatr. Perinat. Epidemiol..

[78] Randriamiharisoa M., Bruyeron O., Boulle Martinaud C., Kraemer K. (2021). Nutri’zaza: Stepping up the provision of sustainable, quality, fortified foods to vulnerable people. Sight and Life: Celebrating Our 35th-Anniversary.

[79] Rousseau S., Steinke J., Vincent M., Andriatseheno H., Pontarollo J. (2023). Strong seasonality in diets and alarming levels of food insecurity and child malnutrition in south-eastern Madagascar. Front. Sustain. Food Syst..

